# Numerical modelling of ^137^Cs content in the pelagic species of the Japanese Pacific coast following the Fukushima Dai-ichi Nuclear Power Plant accident using a size-structured food-web model

**DOI:** 10.1371/journal.pone.0212616

**Published:** 2019-03-13

**Authors:** Mokrane Belharet, Sabine Charmasson, Daisuke Tsumune, Mireille Arnaud, Claude Estournel

**Affiliations:** 1 Institut de Radioprotection et de Sûreté Nucléaire (IRSN), PSE-ENV/SRTE, Laboratoire de Recherche sur les Transferts de radionucléides dans les écosystèmes Aquatiques (LRTA), Saint-Paul Lez Durance, France; 2 Laboratoire d’Aérologie (LA), UMR 5560, CNRS–Université de Toulouse, UPS, Toulouse, France; 3 Environmental Science Research Laboratory, Central Research Institute of Electric Power Industry, Abiko, Japan; University of Maine at Farmington, UNITED STATES

## Abstract

As result of the great east Japan earthquake on March 2011 and the damages of the Fukushima Dai-ichi Nuclear Power Plant (FDNPP), huge amount of radionuclides, especially ^137^Cs, were released to the Japanese Pacific coast. By consequence, several marine species have been contaminated by direct uptake of radionuclides from seawater or through feeding on contaminated preys. In the present study we propose a novel radioecological modelling approach aiming to simulate the radionuclides transfer to pelagic marine species by giving to the organism body-size a key role in the model. We applied the model to estimate the ^137^Cs content in 14 commercially important species of the North-Western Pacific Ocean after the FDNPP accident. Firstly, we validated the model and evaluated its performance using various observed field data, and we demonstrated the importance of using such modelling approach in radioecological studies. Afterwards, we estimated some radioecological metrics, such as the maximum activity concentration, its corresponding time and the ecological half-life, which are important in assessment of the previous, current and future contamination levels of the studied species. Finally, we estimated the time duration required for each species to reach the pre-accident ^137^Cs activity concentrations. The results showed that the contamination levels in the planktivorous species have generally reached the pre-accident levels since about 5 years after the accident (since 2016). While in the case of the higher trophic level species, although the activity concentrations are much lower than the regulatory limit for radiocesium in seafood in Japan (100 Bq kg^-1^), these species still require another 6–14 years (2018–2026) to reach the pre-accident levels.

## Introduction

As result of the great East Japan earthquake on March 11, 2011 and the subsequent tsunami, loss of electric power and hence the failure of the cooling systems of the nuclear reactors of the Fukushima Dai-ichi Nuclear Power Plant (FDNPP) caused the hydrogen explosion of three of them. By consequence, a large amount of radioactive materials entered the marine environment both as fallout and direct releases into the ocean, leading to the contamination of marine species. ^137^Cs and ^134^Cs were released in large quantities with estimates converging on a range of 15−20 PBq for each of them [1]. However, ^137^Cs has a longer half-life time (2.07 yr for ^134^Cs and 30.07yr for ^137^Cs) and will last longer in the environment [1,2].

The released radiocesium reached marine organisms both through uptake from the surrounding water and food ingestion [2]. The results of the monitoring conducted by the Japanese Ministry of Agriculture, Forestry and Fisheries (MAFF) just after the accident, showed that several pelagic species presented ^137^Cs activity concentrations higher than 100 Bq kg^-1^ defined as the regulatory limit for radiocesium in seafood in Japan (limit established on 1^st^ April 2012). However, the high contamination levels characterizing the organisms immediately after the accident were followed by a decreasing trend since summer 2011 [3]. To complete this monitoring process and to understand the dynamics of marine organisms contamination, various modelling studies have been performed [4–8], aiming to both estimate the contamination levels of different organisms following this FDNPP accident and provide more scientific elements helping to understand and interpret the response of marine organisms to the high contamination of their living environments.

The mechanism of radiocesium bioaccumulation by marine organisms is quite complex due to the diversity of contamination sources. For example, a predatory fish takes up radionuclide from water via its gills, and feeds on a variety of prey items belonging to different trophic levels, each of them acquired their contaminant burden via similar mechanisms as the predator [9]. These trophic interactions lead to the contamination of the entire wildlife food web by transfer of the contaminant between the different organisms (from prey to predator). By consequence, food-web bioaccumulation models are the most useful tools to handle this complexity to better characterize and understand the relationship between radionuclide activity concentration in abiotic environmental media and that in the marine organisms.

In the marine ecosystem, size is an important parameter governing biological processes [10]. The use of size spectra to characterize the marine ecosystem is an appealing method to reduce a complex food web to a simple representation [11]. Body size exerts a critical influence on various feeding-related behaviours of individuals, such as predation and predation avoidance, as well as constraining metabolic rate and affecting the rate at which interactions occur between predators and preys [12]. Given that these processes have a substantial effect on the radionuclides transfer between species, and the fact that previous studies have already highlighted the variability of radiocesium content with respect to the organism body size or its position in the trophic chain [13–16], it is important that modern radioecological models be able to involve this fundamental individual-specific trait (the body size) as a critical governing parameter.

In this study, we propose a food-web radioecological modelling approach to simulate the transfer of artificial radionuclides, such as ^137^Cs, to marine pelagic species. We applied this approach to estimate the ^137^Cs activity concentrations in 14 commercially important species of the pelagic food chain of the Japanese Pacific coast, following the FDNPP accident. The purpose of this study was to show the importance of considering the organism body size as a key parameter in the radioecological models, and to better represent the inter- and intra-species variability in terms of ^137^Cs activity concentration due to the size difference between the individuals. A secondary aim was to estimate some radioecological metrics to well understand the contaminant transfer along the trophic chain and to assess the contamination levels of the studied species at different spatial and temporal scales. Finally, we estimated the time duration required for each species to reach the pre-accident activity concentrations.

## Model description and parameterization

In contaminated marine environment, the living organisms accumulate and eliminate simultaneously the radionuclides. For heterotrophs, the radionuclides can be accumulated both from the dissolved phase directly and from ingested food [17]. The transferred radionuclides are, afterwards, eliminated from the organism’s body through biological (excretion and egestion) and physical (radioactive decay) processes.

The dynamics of radionuclides accumulation and elimination by a marine heterotroph organism belonging to species *s* and cohort *a* (a group of individuals from the same age-class) can be expressed by the following equation:

$$\frac{{d[R]}_{s,a}}{dt}=\mu \;{\left[R\right]}_{w}+AE\frac{C_{s,a}}{W_{s,a}}\sum_{i=1}^{N}\sum_{j=1}^{n(i)}\alpha_{i,j}\;{\left[R\right]}_{i,j}-(\lambda_{s,a}+\lambda_{R}+G_{s,a}){\left[R\right]}_{s,a}$$ (1)

where ***[R]***_***s*,*a***_ (*Bq g*^*-1*^) represents the radionuclide activity concentration in the organism, ***[R]***_***i*,*j***_ (*Bq g*^*-1*^) is the radionuclide activity concentration in the prey belonging to species ***i*** and cohort ***j***, ***N*** is the total number of species assumed as preys, ***n(i)*** represents the total number of cohorts related to the prey-species *i*. ***[R]***_***w***_ is the radionuclide activity concentration in seawater (*Bq l*^*-1*^), while ***μ*** (*l g*^*-1*^ *d*^*-1*^) represents the aqueous uptake rate of radionuclides by the organism directly from seawater.

***W***_***s*,*a***_ (*g*) is the weight of the organism, and ***C***_***s*,*a***_ (*g d*^*-1*^) being its daily ration. ***AE*** (dimensionless) is the assimilation efficiency of the radionuclide by the organism, while *α*_*i*,*j*_ ∈ [0,1] (dimensionless) represents the proportion of prey *j* in the diet of the predator *i*.

***λ***_***s*,*a***_ (*d*^*-1*^) represents the biological elimination rate of radionuclide by the organism, ***λ***_***R***_ (*d*^*-1*^) is the rate of the physical radioactive decay of the radionuclide, and ***G***_***s*,*a***_ (*d*^*-1*^) being the organism growth rate.

### Accumulation from water

Uptake of ^137^Cs from water mainly occurs through the gills before being distributed to the whole organism’s body. The uptake rate of radiocesium directly from aqueous phase is reported to be constant and independent of the ambient Cs activity concentration [18]. Due to the lack of precise information about the influence of various environmental or biological factors such as temperature and body size on the ^137^Cs uptake from seawater, we assumed, in this study, a constant uptake rate estimated from the literature, and we speculate that any possible bias generated by this parameter will not grossly impact the findings of this study, since the amount of radiocesium accumulated by the fish directly from water is negligible compared to that accumulated from diet [19].

The uptake rate estimated by [18] for the piscivorous fishes was about *14*.*5 x 10*^*−4*^ *l g*^*-1*^*d*^*-1*^, while [20] reported values ranging from *4 x 10*^*−4*^ to *10 x 10*^*−4*^ *l g*^*-1*^*d*^*-1*^ for the juvenile stage of the sea bream (*Sparus auratus*) exposed to ^134^Cs (which has the same chemical characteristics as ^137^Cs). The value used by [21] and [22] in their models for planktivorous an piscivorous fishes was *0*.*4 x 10*^*−4*^ *l g*^*-1*^*d*^*-1*^. Therefore, the uptake rate value used in this study was *7 x 10*^*−4*^ *l g*^*-1*^*d*^*-1*^ corresponding to the average of these reported values.

### Accumulation from food

Fish must consume enough food to satisfy their energetic requirements for maintenance, growth, activity and reproduction [23]. In contaminated marine environment, the food is generally more or less contaminated leading to the transfer of contaminants from preys to their predators, and the quantity of absorbed radionuclides is mainly depending on the quantity of consumed food and its composition.

The individual-based modelling strategy used in this study to quantify the absorbed radionuclides from ingested food is based on the estimation of: (i) the daily ration of the food consumed by the organism, and (ii) the diet composition (proportion of each prey species in the predator diet).

#### Estimation of the daily food ration

To estimate the daily ration of the food consumed by the organism, we used a simple energy budget equation in which energy consumed by a fish is balanced by total metabolism, waste losses and growth:

Consumption=Metabolism+Wastes+Growth

The equation can therefore be written as:

C=(R+SDA)+(U+F)+G (2)

Where, the metabolic energies *R* and *SDA* are the Respiration and Specific Dynamic Action rates respectively. *F* and *U* represent the waste losses due to egestion (feces) and excretion (nitrogenous wastes) processes, respectively. *G* represents the energy allowed to support organismal growth (growth rate). All the variables represented in this equation are expressed in grams per day (***g d***^***-1***^).

The Respiration rate R is a function of the Standard Metabolic Rate (SMR), an activity factor (R_A_) and a water temperature function:

$$R=(SMR\times R_{A})\times conv\times f(T^{o})$$

where, *conv* represents the coefficient that converts the respiration rate unit from *mg(O*_*2*_*) h*^*-1*^ to *g d*^*-1*^:

$$conv=\frac{434J}{32\;mg(O_{2})}\times \frac{1}{{CAL}_{f}\;{g\;fish}^{-1}}\times \frac{24\;h}{1\;d}$$

and *CAL*_*f*_ is the fish energy density.

The standard (or basal) metabolic rate *SMR* represents the minimum rate of energy expenditure needed to keep a fish alive. It is represented by a simple allometric equation:

$$SMR=a_{R}W^{b_{R}}$$

where the intercept of the allometric equation *a*_*R*_ represents the respiration rate at 0°C of 1 g resting fish. *b*_*R*_ is the slope of the allometric mass function for standard metabolism, and *W* represents the weight of the organism.

The activity factor is represented by an exponential equation, which is function of the fish swimming speed:

$$R_{A}=exp(d_{r}\times V)$$

with *d*_*r*_ being a coefficient relating swimming speed to metabolism (*s m*^*-1*^), and *V* (*m s*^*-1*^) the average swimming speed, which in turn is depending on the fish size *L* (*m*):

*V* = *φ* × *L*, with *φ* (*s*^−1^) being a constant.

The temperature dependence of respiration is represented for the fish species as a simple exponential relationship:

$$f(T^{o})=exp(c_{R}\times T)$$

where T is the water temperature (°C) and $c_{R}=\frac{lnQ_{10}}{10}$ (*Q*_*10*_ the rate at which the function increases over relatively cool water temperature).

The specific dynamic action (SDA) is the daily energetic cost of digestion, assimilation and protein turnover. We formulated SDA as a constant proportion of assimilated energy:

SDA=γ(C−F)

where *γ* represents the specific dynamic action coefficient.

Egestion (*F*) is modelled as a constant proportion of consumption, while excretion (*U*) is formulated as a fraction of assimilated energy:

F=δ×C

U=β(C−F)

where *δ* and *β* are constant probabilities.

With a simple algebraic rearrangement, the equation of consumption ration of an organism belonging to cohort *a* and species *s* can be written as follows:

$$C_{s,a}=\frac{R_{s,a}+G_{s,a}}{1-\eta_{s}}$$ (3)

Where,

$$\eta_{s}=(\gamma +\beta )(1-\delta )+\delta$$

The growth rate is the change over time of the organism body mass. It is expressed as:

$$G_{s,a}=\frac{dW}{da}=\frac{W_{s,a}-W_{s,\left(a-da\right)}}{da}$$ (4)

The fish body mass is related to its length, the length-weight relationship of the fish is worked out as per cubic law given by [24]:

$$W_{s,a}=q\times L_{s,a}{}^{b}$$ (5)

where *L*_*s*,*a*_ is the total fish length (cm) at age *a*, *q* (g cm^-b^) a constant and *b* an exponent usually lying between 2.7 and 3.4 [25].

To estimate the length of studied organisms, we applied the Von-Bertalanffy growth model (VBGM). This model was originally used to describe the relationship between the Age and the Length of the fish assuming a maximum length that might eventually be attained (*L*_∞_). The model is expressed as:

$$L_{s,a}=L_{\infty }\left(1-\exp\left(-k\left(a-a_{0}\right)\right)\right)$$ (6)

where *k* is a constant that defines the rate at which the growth curve approaches the asymptote, *a* is the fish age, and *a*_*0*_ a constant that determines the hypothetical age at which the size of the fish is zero.

The set of ecological parameters used in this model, as well as, their collection sources are summarized in S1 and S2 Tables.

#### Estimation of the organism diet composition

Under nuclear accident conditions, radionuclide activity concentrations in different marine organisms are generally very heterogeneous reflecting the environmental conditions (radiocesium activity concentrations in food and water, temperature, food availability) that could be encountered in their living ecosystem or along their migration trajectories. A good knowledge of the organism diet composition is therefore crucial to properly estimate the quantity of radionuclides ingested by the predator. Usually, radioecological models used constant proportions to represent the diet composition of the organisms [21,22,26], while, in reality, the organism diet is often changing over time with respect to its ontogenetic evolution. Describing the transfer of contaminants between organisms due to feeding interactions is, therefore, challenging as food webs are complex and vary as a function of time and space.

The trophic relationships represent any alimentary relationship involving at least two individuals (predator and prey) belonging to the same ecosystem. The set of these relationships represent the trophic web, which is composed of different trophic levels, and whose base is logically occupied by the primary producers. The trophic interactions between a predator and its different preys can be directed either by a “preferential selection” where the predator selects its preys with respect to their qualities in terms of energy benefits, or by an “opportunistic selection” where the preys are selected only in function of their size and their relative abundance.

In this study, predation is supposed to be totally opportunistic and only derived by the ratio of sizes between organisms. Therefore, all organisms can be potentially predators and preys at the same time, depending on their relative size. To calculate the probability that an organism *i* of size *L*_*i*_ can prey on another organism *j* of size *L*_*j*_, we used the equation reported by [27]

$$S_{i,j}={\left(1+\exp\left(\beta_{1}\left(\rho_{1}-\frac{L_{i}}{L_{j}}\right)\right)\right)}^{-1}*\left(1-{\left(1+\exp\left(\beta_{2}\left(\rho_{2}-\frac{L_{i}}{L_{j}}\right)\right)\right)}^{-1}\right)$$ (7)

**Fig 1:**
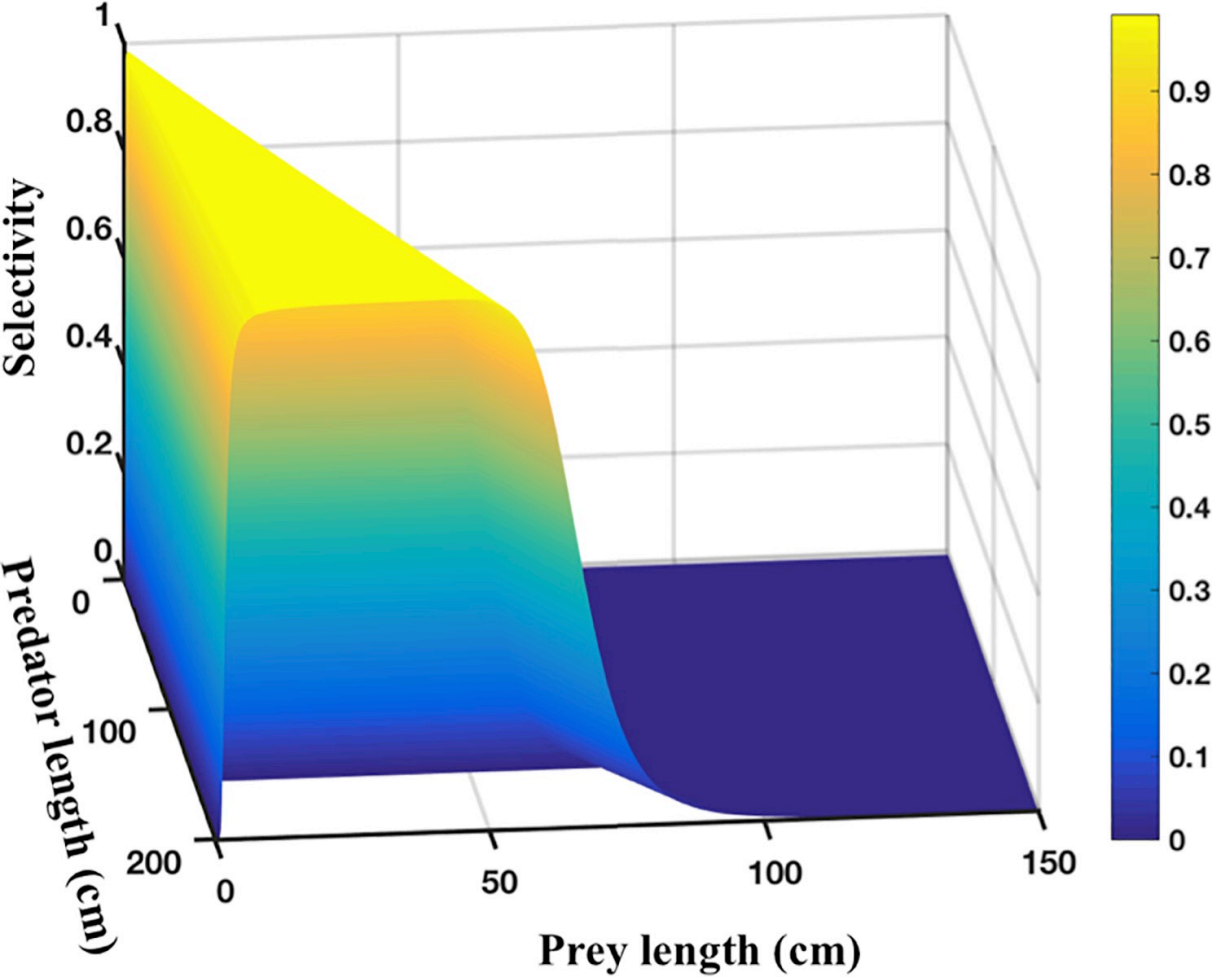
Probability of selectivity versus prey length and predator length, calculated from Eq (7).

With *ρ*_1_, *ρ*_2_, *β*_1_ *and β*_2_, being constant positive parameters characterizing both the half saturation and the flatness of the sigmoid functions (Fig 1).

The diet composition of a predator *i* was estimated by normalizing the probabilities as follows:

$$\alpha_{i,j}=\frac{S_{i,j}}{\sum_{j=1}^{N}S_{i,j}}$$ (8)

The Trophic Levels (TL) of a given individual *i* could be derived from the diet composition as follows:

$$TL_{i}=1+\sum_{j}^{n}TL_{j}*\propto_{i,j}$$ (9)

where *n* being the number of preys *j* assigned to the predator *i*.

### Assimilation efficiency of the radionuclide

Radionuclide Assimilation Efficiency (AE) is defined, here, as the assimilated fraction of the radionuclide contained in the ingested food. Reported ^137^Cs Assimilation efficiency by fish varies between 0.4 and 0.96 [18,20,28,29]. For the purpose of this study, due to the lack of precise information about the variability of this parameter with respect to the fish taxa and/or its size, we assumed a constant Assimilation Efficiency rate (AE) equal to 0.75, as used in previous similar modelling studies [21,22].

### Biological elimination

The ^137^Cs elimination rate from the organism is represented in the model by the biological and the physical elimination rates, as well as, the growth rate that plays a role in the dilution of radionuclide activity concentration in the organism body. For ^137^Cs, the physical half-life is fairly high (30 years), leading to a negligible physical elimination rate compared to the biological elimination.

**Fig 2:**
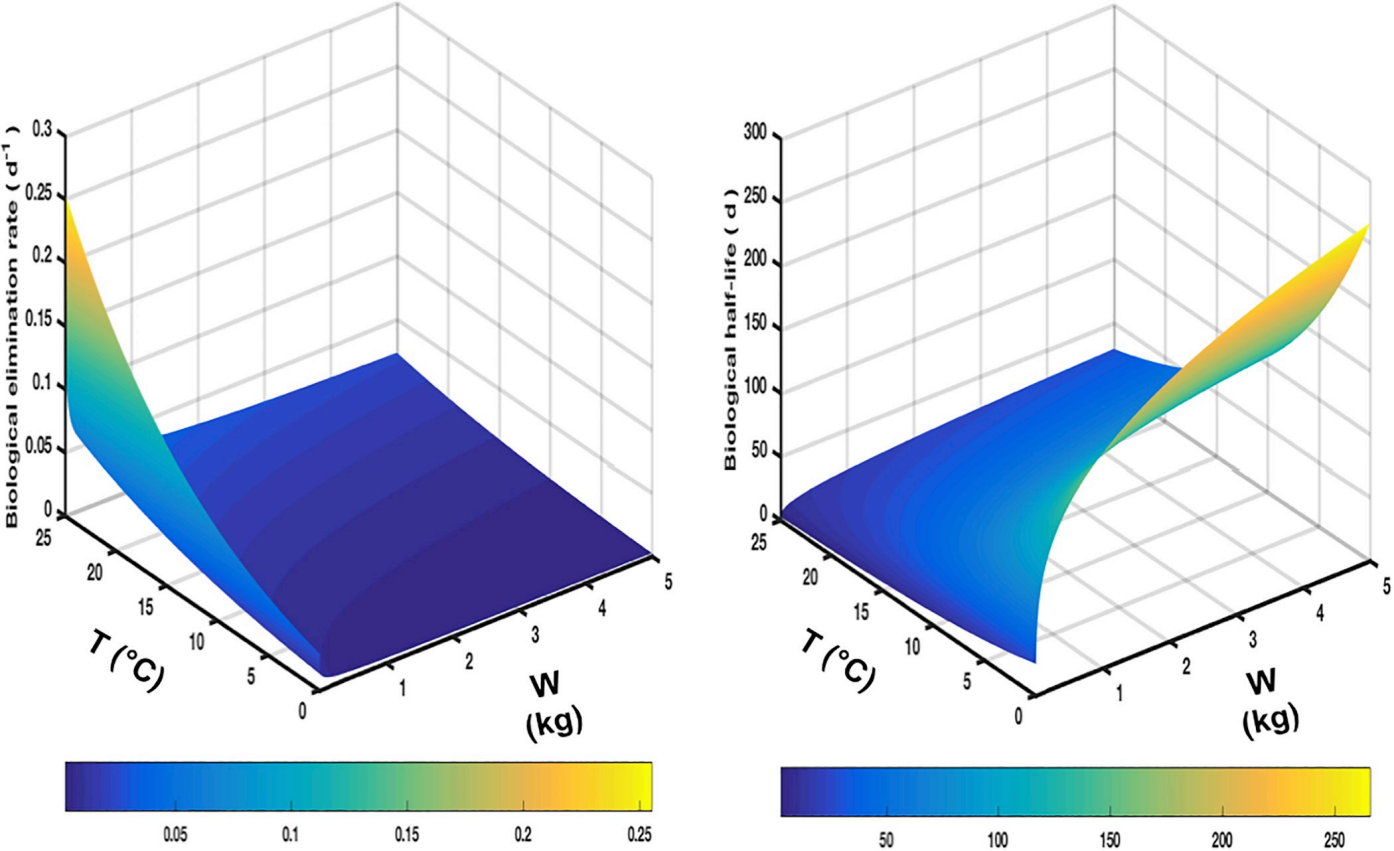
Variability of the biological elimination rate (left) calculated using Eq (10) and the biological half-life (right), with the water temperature and the organism weight.

To estimate the ^137^Cs biological elimination rate in this study, we used the allometric relationship reported in [30]. This relation estimates the elimination rate as a function of the organism weight and the water temperature (Fig 2), and can be written, after rearrangements, as follows:

$$\lambda_{s,a}={W_{s,a}}^{-0.28}*10^{\left(9.03-\frac{0.27}{kT}\right)}$$ (10)

with *k* being the Boltzmann constant *(~ 8*.62 * 10^*−5*^ *eV K*^*-1*^), and T the water temperature.

## Model application

### Study area

**Fig 3:**
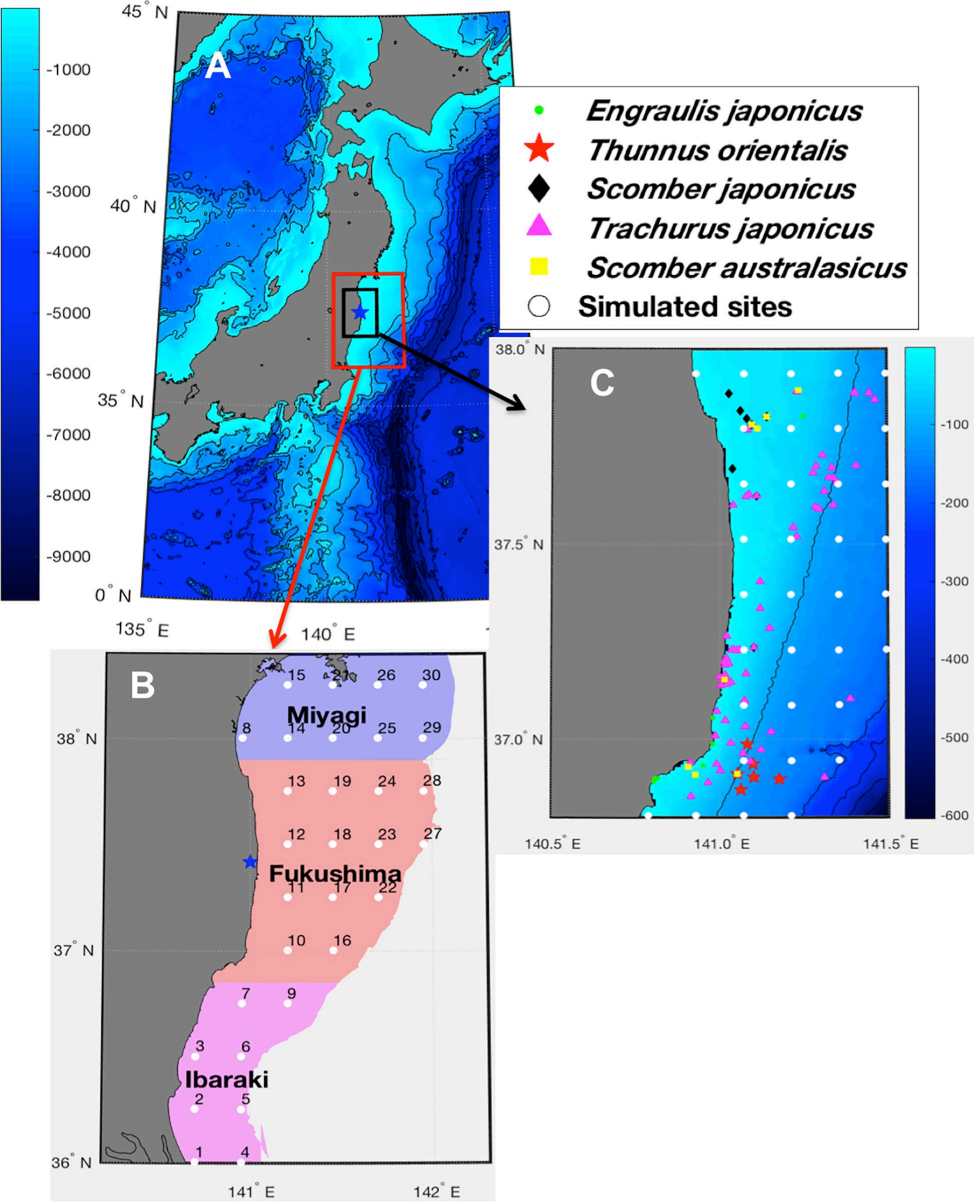
(A) Map of Japan showing the location of the study area in the Pacific coast. (B) Positions of the 30 simulated sites (numbered from 1 to 30) used for the validation of the model in the case of the non-georeferenced data. (C) zoom of the area with a higher spatial resolution, showing the positions of the sites used for the validation of the model in the case of the georeferenced data.

Our study area covers the eastern coast of Japan located between 36 and 38.5°N, and between the shore and the isobath 500 m (Fig 3A). This area was subdivided into 30 evenly spaced sites (Fig 3B) to take into account the spacial variability of ^137^Cs activity concentration in seawater and of water temperature. The distance between two successive is 0.25° (about 28 km). However, a large part of the data available for validation is located in the area between 36.8°– 38° N and between 0–200 m depth. Therefore, for the purpose of model validation and for more precision, the spatial resolution in this coastal area was increased (about 0.11°) as shown in Fig 3C.

### Studied species

**Fig 4:**
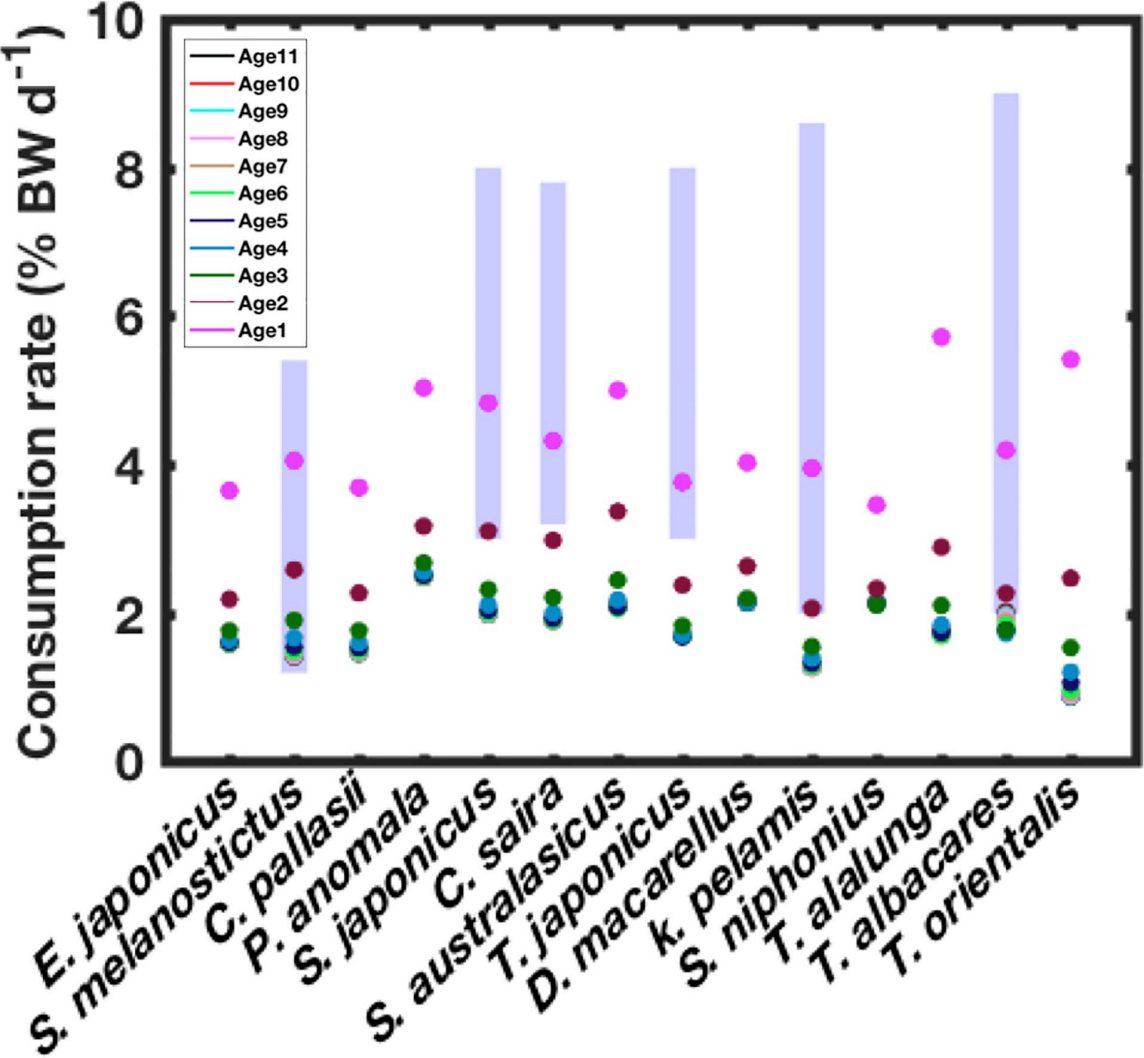
Estimated consumption rates for the 14 studied species, confronted with the variability ranges of the consumption rates reported in the literature (blue bars). The colours of the dots correspond to the different cohorts (2002–2013). The observed data are collected from the references [39–50]. The studied species are shown in an increasing order of their asymptotic maximum Length.

The studied ecosystem consists of 14 pelagic marine species ranging from the small planktivorous fishes such as sardine and anchovy to the higher predatory fishes such as the tuna species. These species are of great importance in this region in terms of abundance and/or commercial interests. The considered species are shown in Fig 4 and listed in Table 1 by their scientific and common names.

**Table 1:** List of the 14 pelagic fish species considered in this study.

Scientific name	Common name
*Engraulis japonicus*	Japanese anchovy
*Sardinops melanostictus*	Japanese sardine
*Clupea pallasii*	Pacific herring
*Psenopsis anomala*	Rudderfish
*Scomber japonicus*	Chub mackerel
*Cololabis saira*	Pacific saury
*Scomber australisicus*	Spotted mackerel
*Trachurus japonicus*	Jack mackerel
*Decapterus macarellus*	Mackerel scad
*Katsuwonus pelamis*	Skipjack tuna
*Scomberomorus niphonius*	Japanese Spanish mackerel
*Thunnus alalunga*	Albacore
*Thunnus albacares*	Yellowfin tuna
*Thunnus orientalis*	Bluefin tuna

For each species, one cohort by year was considered taking into account the spawning period. The spawning dates corresponding to the starting date for each cohort were randomly selected from a normal distribution in which the mean value represents the date at which the spawning is generally at the highest.

### Numerical simulations

Under the assumption of a limited movement of the studied species (the species are assumed to keep their position throughout the simulation period), we carried out 11 years of simulation from January 2002 to February 2013, assuming a time step of 1 day chosen to take into account the high temporal variability of ^137^Cs activity concentration in seawater after the accident.

During the last decade before the accident (11 March 2011), the ^137^Cs activity concentrations in the North Western Pacific (NWP) Ocean ranged from 1 to 3 *x 10*^*−3*^ *Bq l*^*-3*^ [31,32]. Therefore, the ^137^Cs activity concentrations in seawater used in the simulations during this period were randomly selected from a uniform distribution within the interval *[1, 3] x 10*^*−3*^ *Bq l*^*-1*^.

After the accident we used the results of the numerical simulation of ^137^Cs distribution in the NWP Ocean carried out by [33], extended to cover our simulation period [34]. To simplify, and for the purpose of the modelling, we considered in this study only one group of phytoplankton with size ranging from 0.02 μm to 2 mm and only one group of zooplankton with size ranging from 0.2 mm to 3 cm. The ^137^Cs activity concentrations in these two groups (*Bq g*^*-1*^) are calculated using their activity concentration ratios derived from the simulation results reported by [35] and the ^137^Cs activity concentration in seawater as follow:

$${{[}^{137}Cs]}_{pk}=CR_{pk}\times {{[}^{137}Cs]}_{w}$$

$${{[}^{137}Cs]}_{zk}=CR_{zk}\times {{[}^{137}Cs]}_{w}$$

*CR*_*pk*_ represents the weighted mean of the activity concentration ratios obtained for the 2 groups of phytoplankton (Small phytoplankton PS and Large phytoplankton PL), and *CR*_*zk*_ is the weighted mean of the activity concentration ratios estimated for the 3 groups of zooplankton (Small zooplankton ZS, Large zooplankton ZL and Predatory zooplankton ZP). For more details see [35].

For the purpose of this study, we used daily average seawater temperature data at ¼ degree horizontal resolution obtained from the global ocean physics reanalysis generated by the NEMO ocean model [36]. These data were downloaded directly from the Copernicus marine environment monitoring service (www.marine.copernicus.eu) over the period from January 2002 to February 2011. From March 2011, we used the same temperature data used by [33] extracted from JCOPE2 reanalysis data (Japan Coastal Ocean Prediction Experiment 2, [37]).

### Field data

To validate the model and to test its performance, we used two sources of field data: (1) the georeferenced data for which the geographic coordinates of the place where the fish was caught are indicated, and (2) the non-georeferenced data for which only the prefecture corresponding to the place where the fish was caught are indicated without any information about the precise geographic coordinates. For both types of data, no information on the size of sampled organisms was provided.

The non-georeferenced and a part of georeferenced data were collected from the website of the Japanese Fisheries Agency of Ministry of Agriculture, Forestry and Fisheries (http://www.jfa.maff.go.jp/e/inspection/index.html), which regularly published the results of the monitoring on radioactive materials in fishery products since March 23^rd^, 2011. The other part of the georeferenced data was extracted from the data reported by [38].

In this study, all the field data and the simulation results are expressed in *Bq kg*^*-1*^ *wet-weight*.

## Results

### Consumption rates and trophic levels

Estimated consumption rates, using Eq (3) for the species-related cohorts are presented in Fig 4. The few available observation data collected from the literature for some species are also presented as ranges of variability.

The estimated values vary from one species to another and within the same species (between the different cohorts). These estimations range from 1 to 6% BW (Body weight) per day. They are, in general, close to the observed data reported in the literature [39–50], especially in the case of the Japanese sardine (*S*. *melanostictus*) and the Japanese saury (*C*. *saira*).

**Fig 5:**
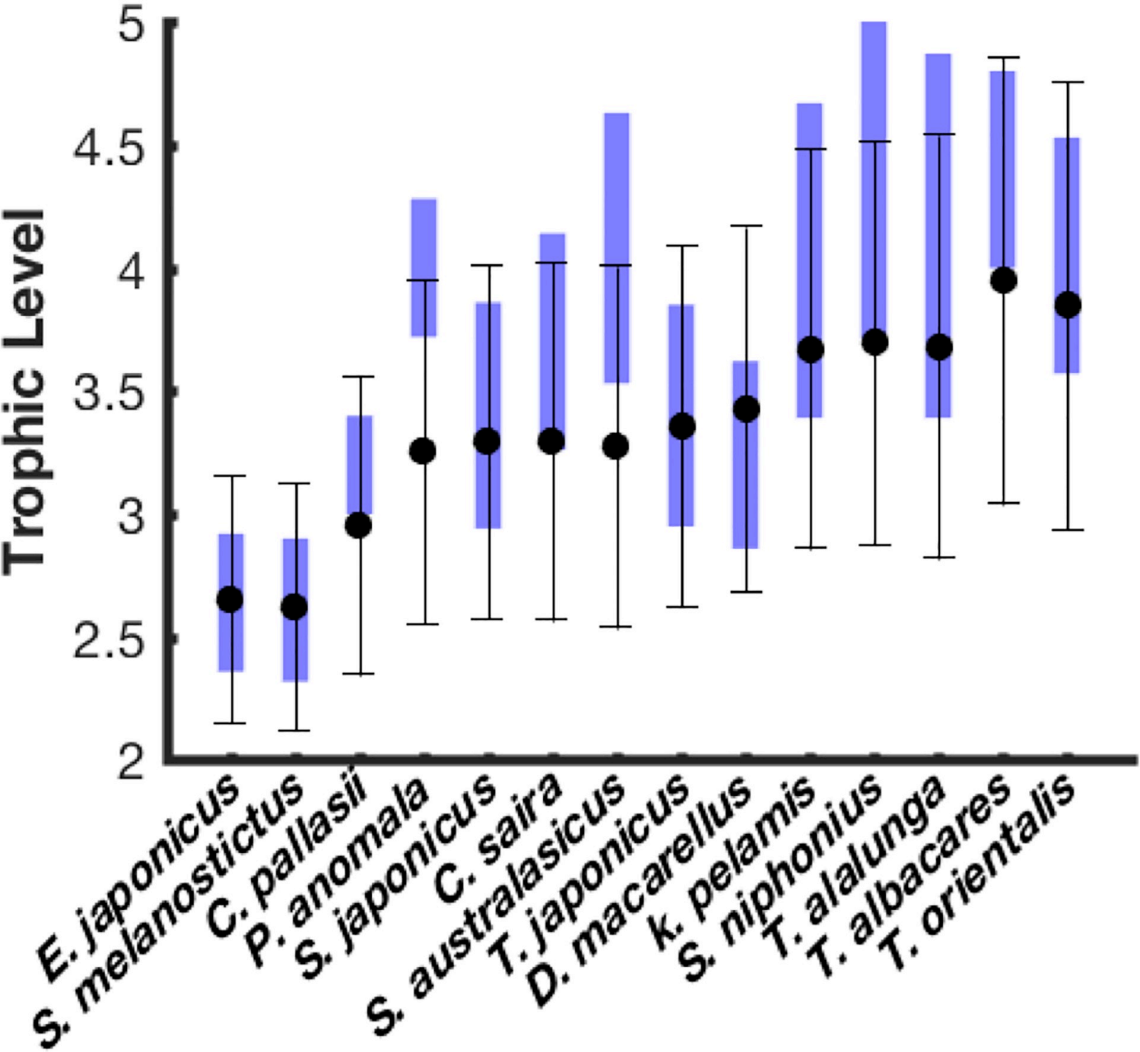
Comparison between the estimated trophic levels and the observed values for the 14 studied species. Black error bars represent the intra-species variability (averages and standard deviation) calculated over all the species-related cohorts. The blue bars are the reported observations in Fishbase database. The studied species are shown in an increasing order of their asymptotic maximum Length.

The trophic levels estimated for the studied species at the end of 2012 using the Eq (9) are shown in Fig 5. The estimated values are in general consistent with the observed data reported in the literature (Fishbase website). The small differences may be ascribed to the simplicity of our approach that assumed an ecosystem of only 14 species, while in reality the pelagic ecosystem is much more complex and may contain many other species able to be part of the organism diet. Added to that, the fact that in our approach we considered that the predation is fully opportunistic and is only determined by the predator/prey size ratio, while in the nature, some species may be selective by presenting certain preferences towards some specific preys.

In general, the results obtained in this study for both consumption rates and diet compositions were satisfactory, and we speculate that the small differences observed between the field data and simulation results would not significantly impact the findings of this study.

### Model validation

#### Pre-accident period

The field data related to the ^137^Cs activity concentration in fish before the accident are very scarce and are not available for all the studied species. The validation process involving all these species is, therefore, not possible.

**Fig 6:**
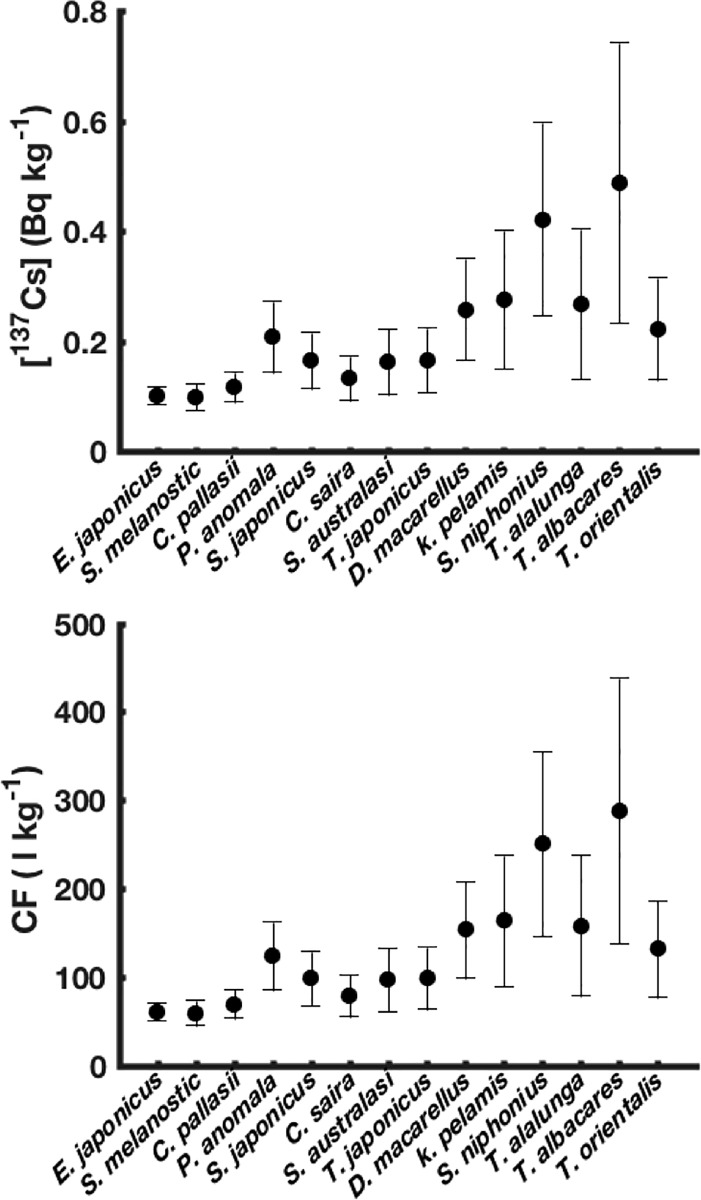
Simulated ^137^Cs activity concentrations (Bq kg^-1^ wet weight) and concentration factors (l kg^-1^) for the 14 studied species in the steady state conditions before the accident. The studied species are shown in an increasing order of their asymptotic maximum Length.

Values of simulated ^137^Cs activity concentrations and Concentration Factor (ratio between ^137^Cs activity concentration in fish and its activity concentration in seawater) for the pre-accident period are shown in Fig 6 for the 14 studied species. The mean and standard deviation represented for each species are calculated on the 1^st^ of March 2011 involving all the species-related cohorts throughout our study area. The standard deviation corresponds to the intra-species variability (the variability between the different cohorts of each species).

The simulated ^137^Cs activity concentrations for the different species range from about 0.*10*±*0*.*05 Bq kg*^*-1*^ in the case of small pelagic species such as the Japanese sardine (*S*. *melanostictus*) to about *0*.*49*±*0*.*25 Bq kg*^*-1*^ for large predators such as the Yellowfin tuna (*T*. *albacares*). The high standard deviation calculated for the longest fish, reflecting the higher intra-species variability, compared to the smaller species, is due to the fact that, for the longest species, the size difference between the individuals belonging to the different cohorts is quite high, leading to a clear difference between them in terms of diet composition and biological elimination rates. In the case of the small pelagic species, however, the size difference between the cohorts is small and the diet composition is globally not different (plankton), leading to very small intra-species variability.

[51] reported a time series of ^137^Cs activity concentrations in skipjack tuna (*K*. *pelamis*) between 2000 and 2010. At the end of this time series (one year before the accident), the activity concentrations were about *0*.*15–0*.*20 Bq kg*^*-1*^ and are, therefore, in good agreement with our finding regarding this species (0.27 ±*0*.*12)*.

For some species the predicted values are slightly higher than observed ones. The observed ^137^Cs activity concentrations in the rudderfish *P*. *anomala* during the period 1984–1995 are between *0*.*10* and *0*.*26 Bq kg*^*-1*^ with an observed ecological half-life of about 8.6 years [14]. In 2011 (just before the accident), these observed activity concentrations should be ranging between about *0*.*05* and *0*.*12 Bq kg*^*-1*^ (when applying the observed ecological half-life of 8.6 years for a period of 10 years), and are therefore two times lower than the predicted values (0.21 ±*0*.*06 Bq kg*^*-1-*^). The predicted value of ^137^Cs activity concentration in Japanese sardine *S*. *melanostictus* is *0*.*10* ± *0*.*02 Bq kg*^*-1*^, which is about two times higher than the observed activity concentrations (i.e. *0*.*05* ±*0*.*02 Bq kg*^*-1*^) in one species of the same gender (*Sardinops sp*.) in the Japanese coasts [52].

The simulated Concentration Factors (CF) are ranging between *60* ±*14 l kg*^*-1*^ and *287* ±*150 l kg*^*-1*^ depending upon the species (Fig 6). The minimum value is estimated for the Japanese sardine (*S*. *melanostictus*), while the maximum value is found for the Yellowfin tuna (*T*. *albacares*). The simulated CF for the small pelagic fishes range between about *55* and *124 l kg*^*-1*^, and are therefore in good agreement with the CF reported by [53] for some Japanese coastal fishes over the period 1984–1990, *i*.*e*. from *14* to *133 l kg*^*-1*^. The average value of the simulated CF for the small pelagic fish is about *93 l kg*^*-1*^, which therefore well match with the International Atomic Energy Agency recommended value for the marine pelagic fish, which is 100 *l kg*^*-1*^ [54].

#### Post-accident period

**Fig 7:**
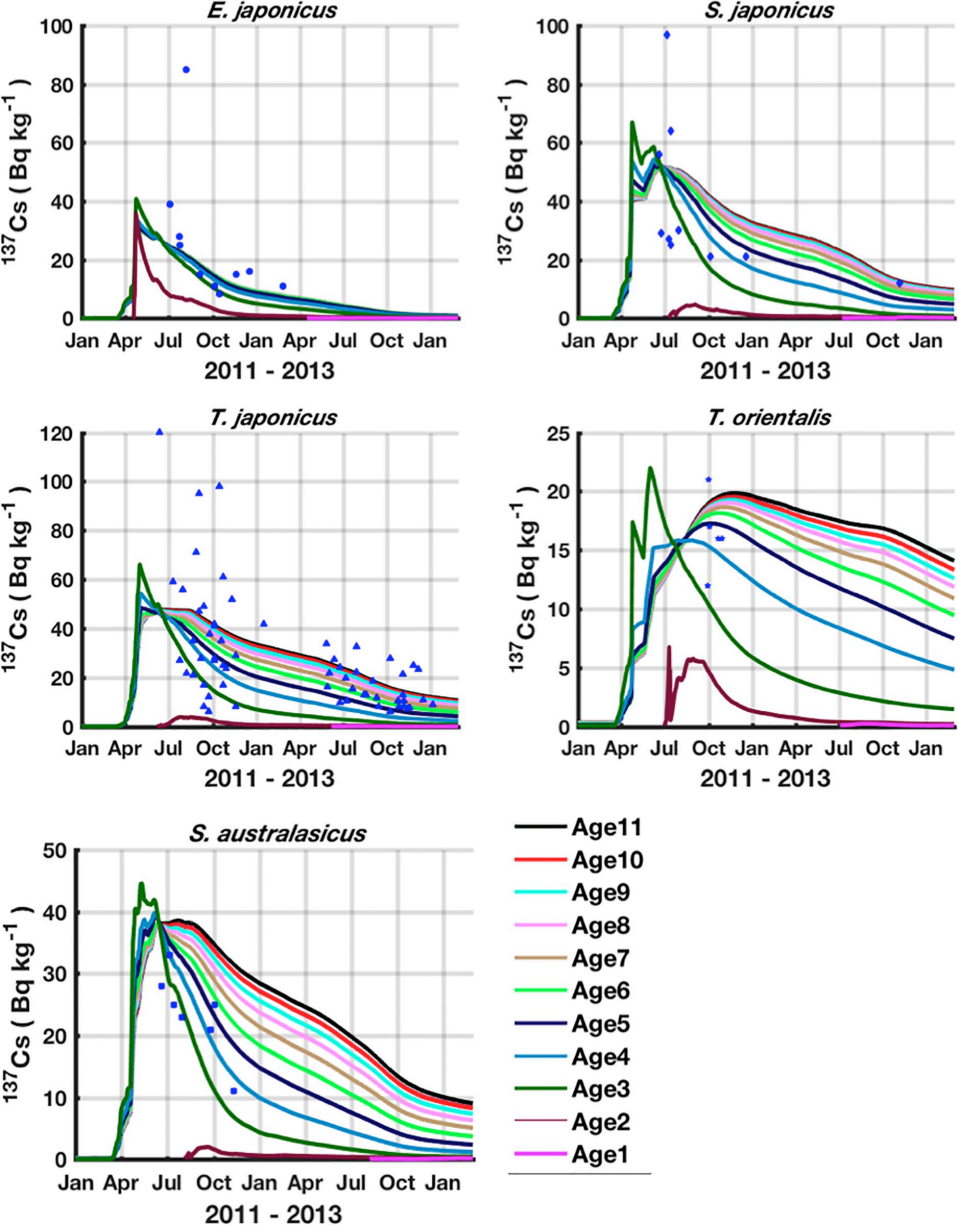
Time series of the predicted ^137^Cs activity concentrations (Bq kg^-1^ wet weight) in the different cohorts (individuals born in the same year), confronted with the observed field data (coloured symbols) of 5 studied species (for which the data are available). For each species, 11 cohorts are represented (Age1 includes individuals aged from 0 to 1 years, Age2 includes individuals aged from 1 to 2 years, etc). Each curve represents the dynamics of ^137^Cs activity concentration in only one cohort. For each cohort, the shown ^137^Cs activity concentration corresponds to the median of the 35 sites of the high spatial resolution area (see Fig 3C).

**Fig 8:**
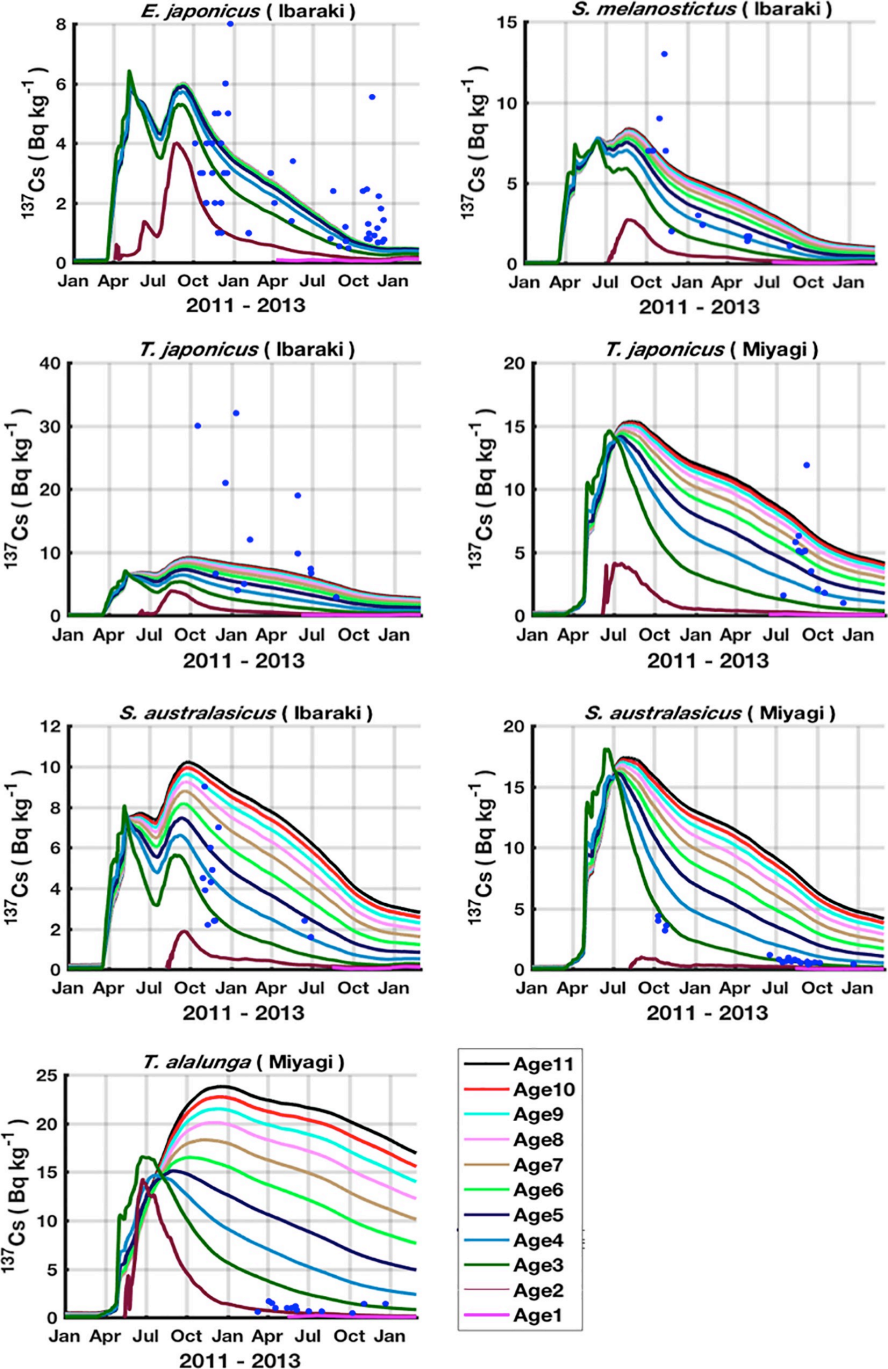
Time series of the predicted ^137^Cs activity concentrations (Bq kg^-1^ wet weight) in the different cohorts (coloured curves), confronted with the observed field data (coloured symbols). For each species, 11 cohorts are represented (Age1 includes individuals aged from 0 to 1 years, Age2 includes individuals aged from 1 to 2 years, etc). Each curve represents the dynamics of ^137^Cs activity concentration in only one cohort. For each cohort, the shown ^137^Cs activity concentration corresponds to the median of the sites belonging to the prefecture in which the observed individuals are caught (see Fig 3B).

Temporal dynamics of ^137^Cs activity concentrations in the studied species for which the observation field data are available are shown in Figs 7 and 8 for the period from Jan 2011 to Feb 2013. The simulation results are represented for all the species-related cohorts and are confronted with observed field data. In the Fig 7 each cohort curve illustrates the spatial mean of the highest spatial resolution area, while in Fig 8, each cohort curve corresponds to the spatial average in the prefecture to which the field data are assigned (see Fig 3). Due to the lack of information about the size of the observed fish species, the observations are confronted with the simulation results of all the species-related cohorts.

**Fig 9:**
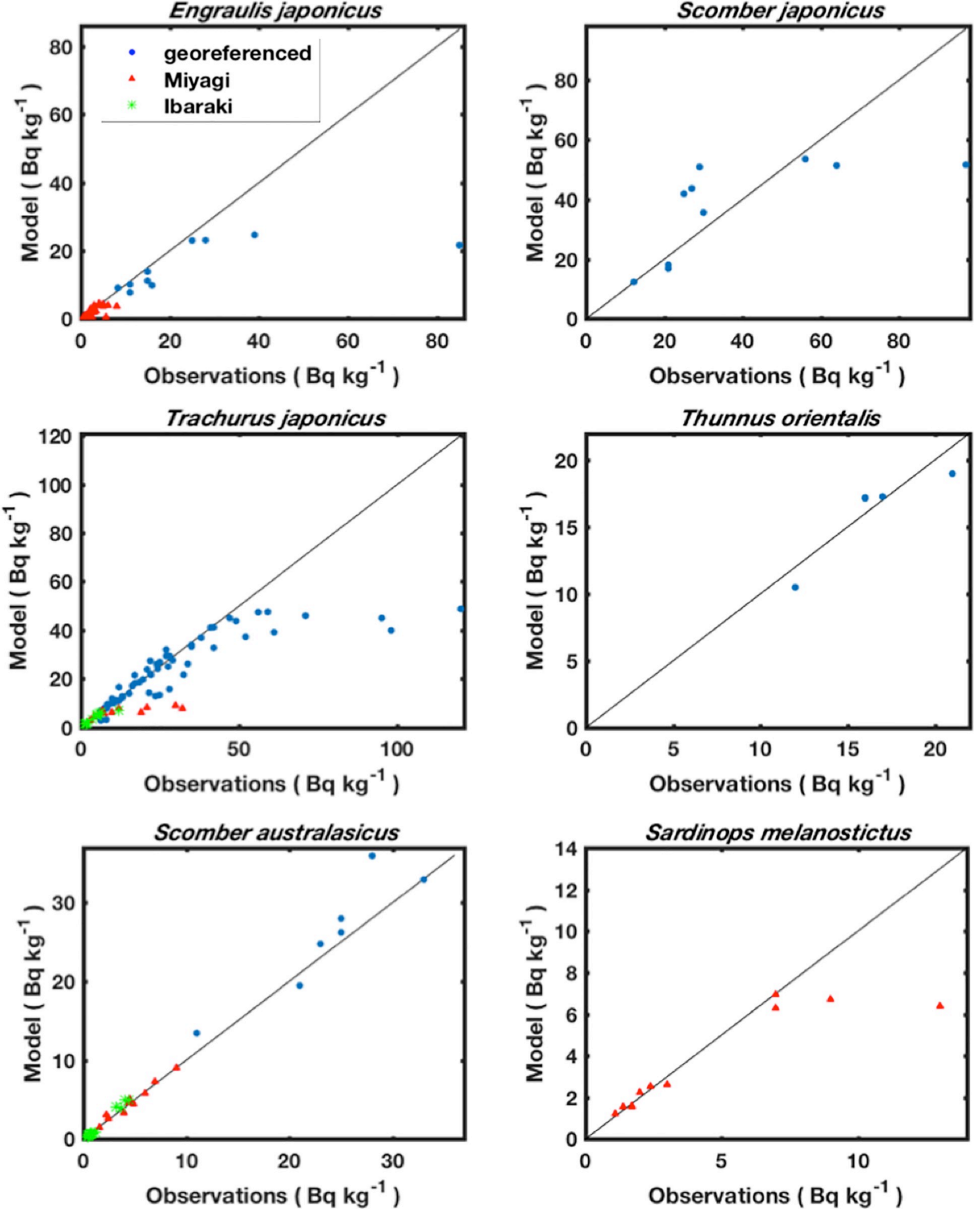
Predicted versus observed ^137^Cs activity concentrations (Bq kg^-1^) in 6 species shown in Figs 7 and 8.

Comparisons between observed and simulated ^137^Cs activity concentrations are shown in Fig 9. The results show a good agreement between observed and simulated activity concentrations. This finding means that our radioecological model successfully predicts the contamination levels observed in the marine pelagic species in the 2 years following the FDNPP accident.

### Radioecological metrics

**Fig 10:**
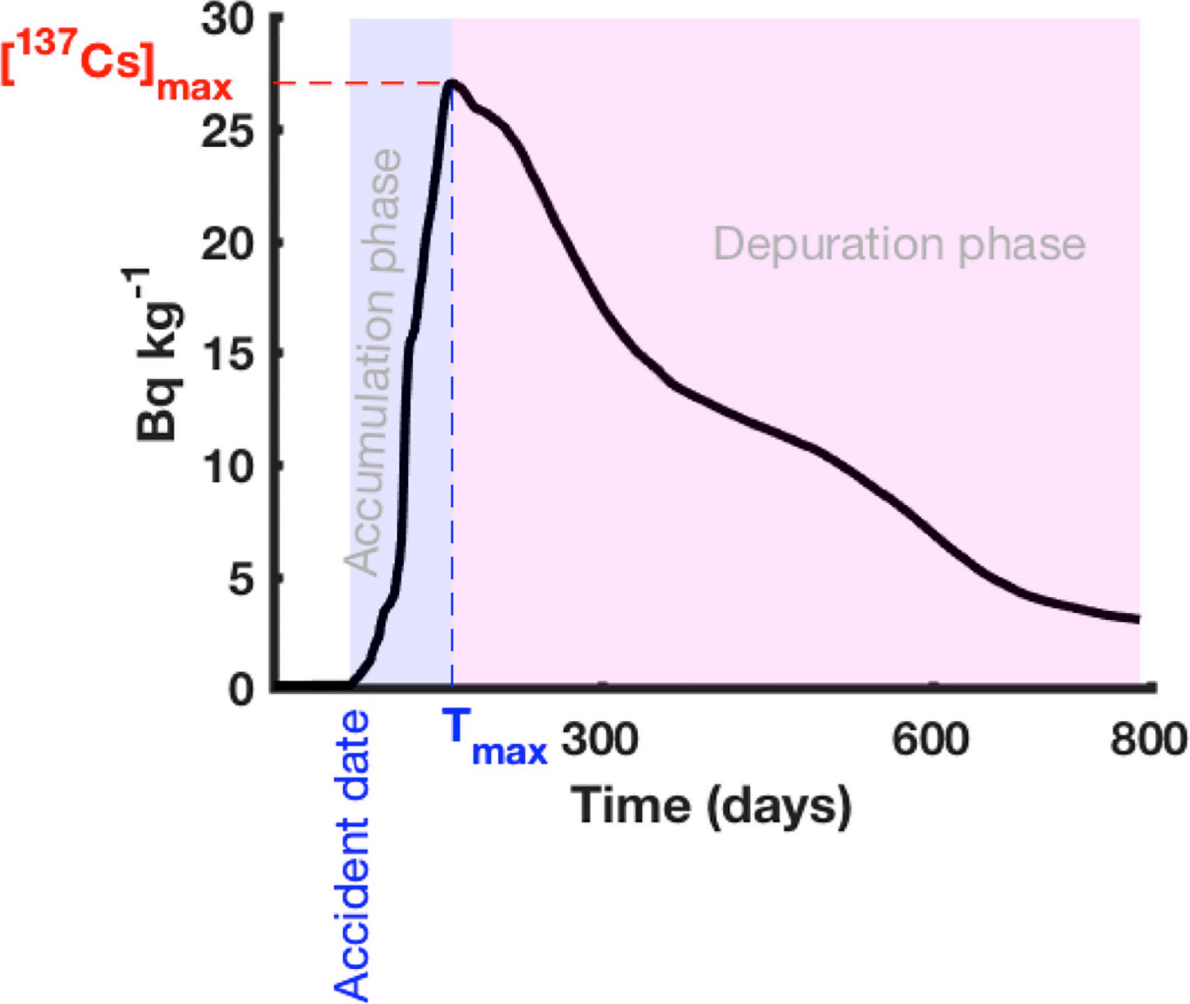
Typical curve of the ^137^Cs activity concentration dynamics in the marine organism, illustrating the accumulation and elimination phases as well as the different radioecological metrics.

After an accidental situation with a high radioactive contamination of the seawater, the shape of the curve representing the dynamics of the level of contamination in marine organisms generally resembles that shown in Fig 10. Although the accumulation and elimination processes occur simultaneously, the predominance of one of the two determines the shape of the curve. This curve is characterized by two successive phases separated by *T*_*max*_ corresponding to the time, from the beginning of the contamination process, at which the ^137^Cs activity concentration in the marine organism reaches its maximum value. These two phases are: (1) the accumulation phase that corresponds to the period over which the accumulation process predominates, leading to a rapid increase of the contaminant activity concentration in the organism; and (2) the elimination phase that represents the period over which the elimination process predominates, leading to a progressive decrease of the ^137^Cs activity concentration in the organism.

#### Maximal activity concentrations

**Fig 11:**
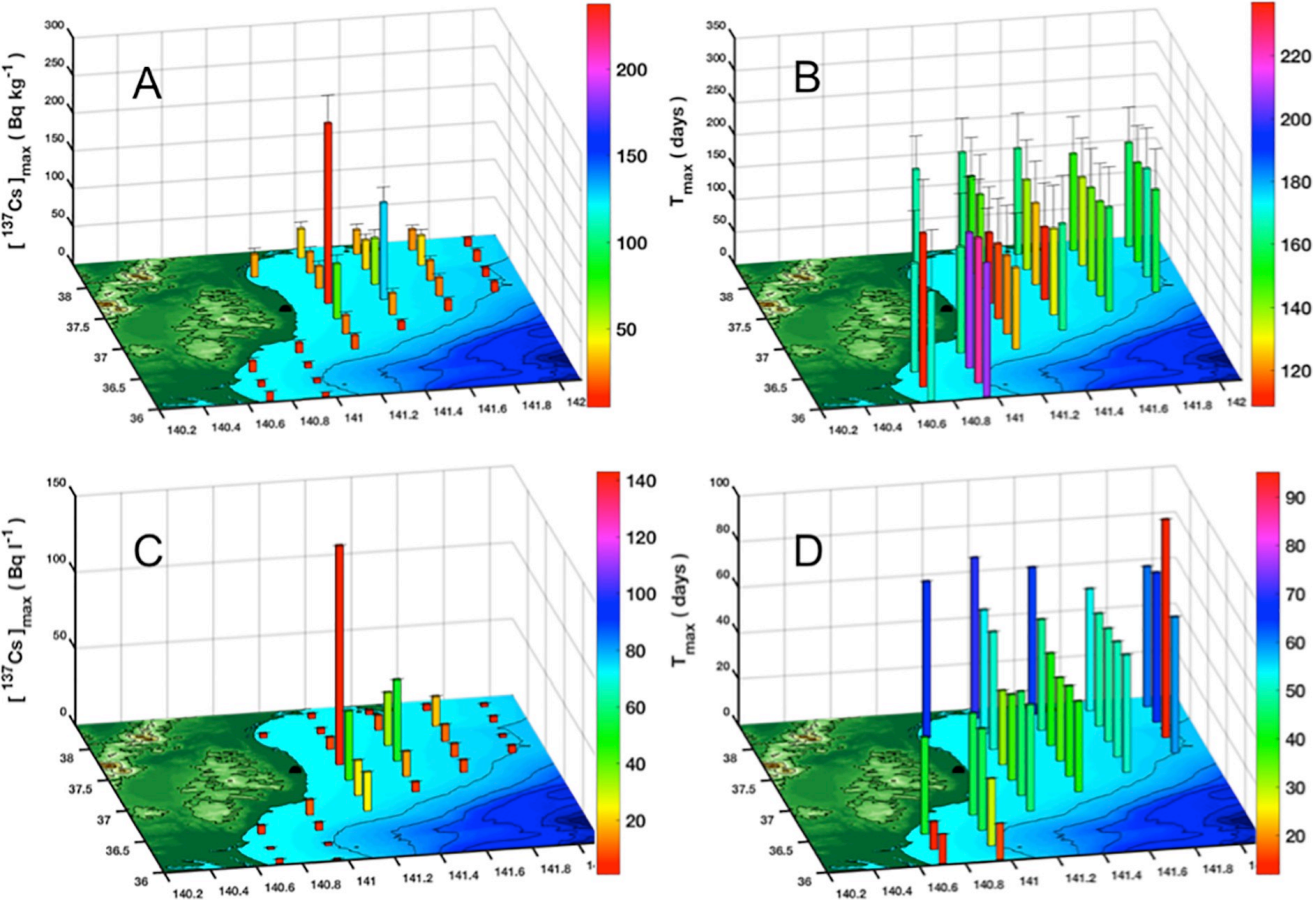
Spatial distribution of: (A) the maximum ^137^Cs activity concentration in the organism (Bq kg^-1^), (B) its corresponding T_max_ (in days), (C) the maximum ^137^Cs activity concentration in seawater (in Bq l^-1^), (D) the T_max_ corresponding to the maximum concentration in seawater (in days). The values shown in this Figure are calculated over the cohorts related to all the studied species.

The spatial distribution of mean and standard deviation values of the maximum ^137^Cs activity concentrations in the different species-related cohorts is represented in Fig 11A. The values change with respect to the location, and range between *4*.*7*± *1*.*5 Bq kg*^*-1*^ (found at site 5) and *238*.*0*± *36*.*5 Bq kg*^*-1*^ (in front of FDNPP at site 12). This spatial distribution is similar to that of the maximum activity concentration of ^137^Cs in seawater (Fig 11C). The high activity concentrations estimated in front of FDNPP (sites 11, 12 and 18) are mainly due to the strong confinement of the contaminated waters in the vicinity of FDNPP during the high input period [33,55]. Furthermore, the maximum activity concentrations found in the northern part (Miyagi prefecture) are, in general, higher than those found in the southern part (Ibaraki prefecture), although the maximum ^137^Cs activity concentrations in seawater were almost similar in the two parts (Fig 11C). This can be explained by the residence time of the contaminated waters, which is higher in the northern sites compared to the southern ones (see S1 Fig).

**Fig 12:**
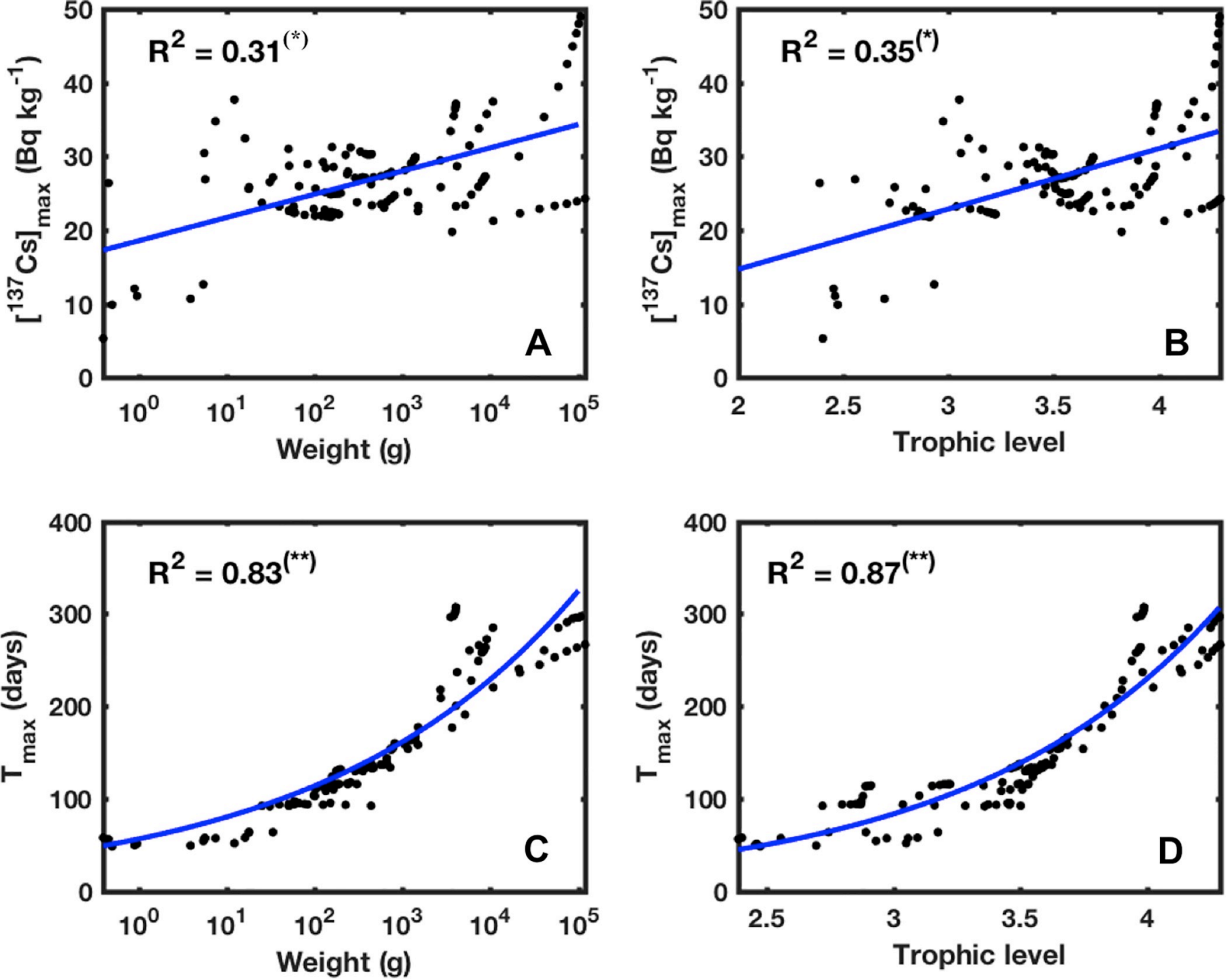
Predicted maximum activity concentrations (in Bq kg^-1^) and T_max_ (in days) of the species-related cohorts, plotted as a function of the their weights and trophic levels. Significance level of the correlation: p>0.05 (), p<0.05 (*), p<0.01 (**).

In Fig 12A and 12B the mean values of the maximum ^137^Cs activity concentrations calculated for the species-related cohorts, are plotted as function of their corresponding Weights and estimated Trophic Levels respectively. These figures show moderate positive linear correlations between the maximum ^137^Cs activity concentrations in the organism and the corresponding Weight and TL. This correlation is less good especially when the organism weights about 10 to 100 g (or 3 < TL < 3.7), showing a steady trend rather than an upward trend.

#### Duration of the accumulation phase

Fig 11B and 11D show, respectively, the spatial distribution of *T*_*max*_ averaged over the 14 studied species, and the spatial distribution of *T*_*max*_ corresponding to the maximum ^137^Cs activity concentration in seawater. The species-related *T*_*max*_ range between 109 and 237 days, while the seawater-related *T*_*max*_ vary between 12 and 95 days depending upon the site location. The spatial distribution of species-related *T*_*max*_ shows highest values in Miyagi prefecture sites (especially sites 2, 4, 5 and 6) although the seawater-related *T*_*max*_ in the same sites is the lowest. This can be explained by the presence, in these sites, of a second lower peak that arrived some days after the first one (see S1 Fig) extending, thereby, the accumulation process and delaying the peak of the ^137^Cs activity concentration in the organisms. In the rest of the study area, the spatial distribution of the species-related *T*_*max*_ is almost the same as that of seawater-related *T*_*max*_.

For each species, the cohorts-related *T*_*max*_ are averaged over the whole study area and are plotted as functions of their corresponding weight (Fig 12C) and estimated Trophic Levels (Fig 12D). These figures show significant one-term exponential correlations with high coefficients of determination (R^2^), indicating a very good correlation between *T*_*max*_ and Weight and/or TL. The growing exponential trend shows that *T*_*max*_ estimated for the small organisms is significantly lower compared to the larger ones. This can be explained by the difference, in terms of diet composition, between these organisms in the sense that the smaller ones feed mainly on plankton, which requires only short time to reach equilibrium with seawater [35], leading to shorter *T*_*max*_ (i.e. shorter accumulation phase). While in the case of larger organisms, the diet composition is more diverse and contains large individuals that require longer time to reach their maximum activity concentrations, leading to an extension of the accumulation period and thus to longer *T*_*max*_.

#### The elimination phase

**Fig 13:**
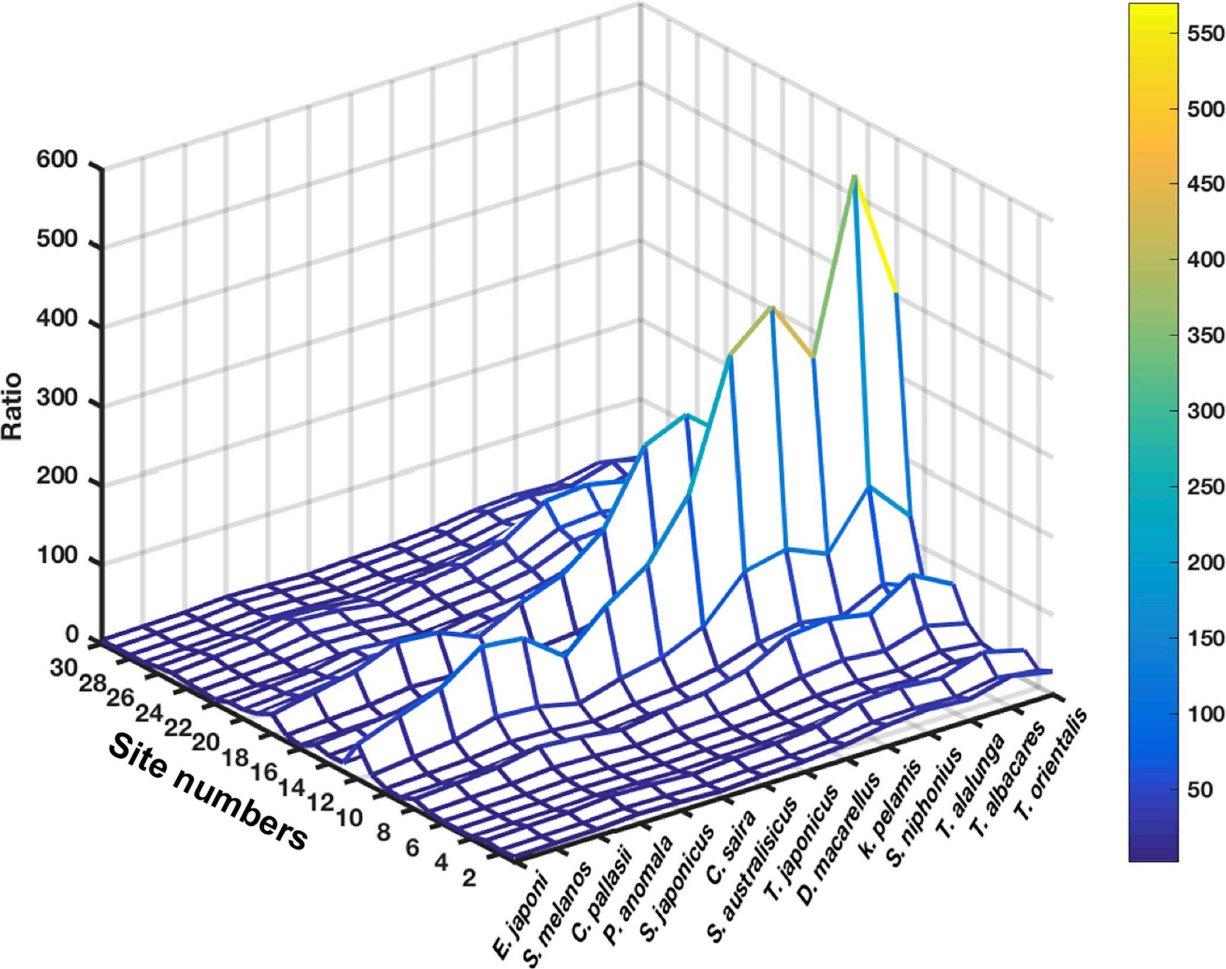
Ratio between the ^137^Cs activity concentration in the organism at the end of the simulation (28 Feb 2013) and the activity concentration in the same organism two years before (28 Feb 2011), shown as function of studied species (x-axis) and sites (y-axis). The studied species are shown in an increasing order of their asymptotic lengths.

Fig 13 exhibits, for the simulated species and sites, the ratio between ^137^Cs activity concentrations at the end of the simulation period (Feb 2013) and those just before the accident (March 2011). For each species, the mean value of its related cohorts is shown in the figure. The predicted ratios vary from 2.38± 0.03 for the Japanese Anchovy *E*. *japonicus* at site 4 to about 570±142 at site 12 for the Yellowfin tuna *T*. *albacares*, meaning that, in the extreme situations (if the species movements are very limited throughout the period of the highest contamination of seawater), ^137^Cs activity concentrations in the pelagic species, two years after the accident, might be more than 2 orders of magnitude higher than its activity concentrations before the accident, depending upon species and sites. The highest values for the studied species are mainly found at the sites located close to the damaged FDNPP (especially 12, 18, 11), in which the inter-species variability is also the highest. The results show also that, predicted ratios are generally increasing with the asymptotic maximum Length of the studied species (the studied species are shown in an increasing order of their asymptotic Lengths). This trend is most noticeable in the sites with the highest contamination levels (the closest to the FDNPP).

**Fig 14:**
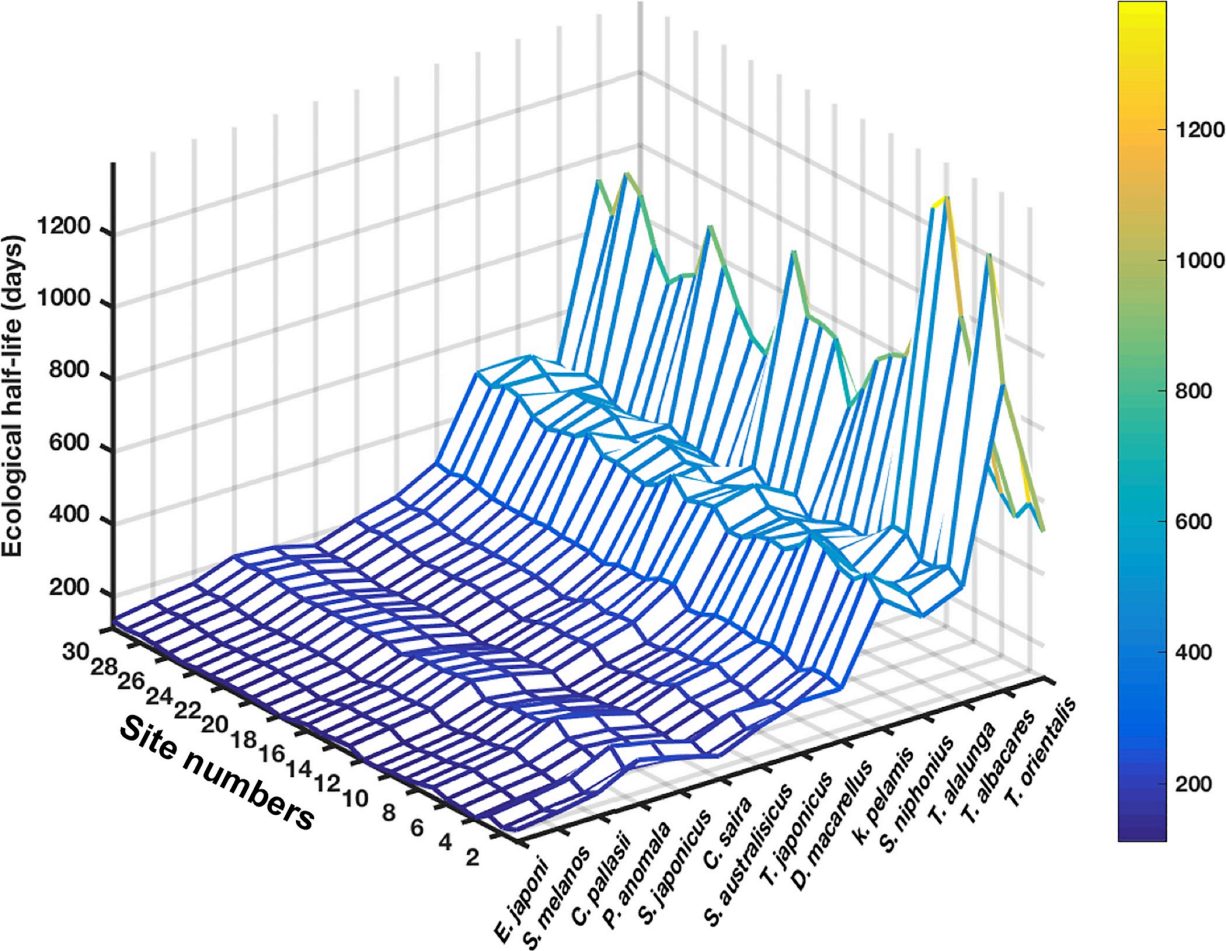
Estimated ecological half-lives (in days) shown as function of studied species (x-axis) and sites (y-axis). The studied species are shown in an increasing order of their asymptotic Lengths.

To measure the decreasing speed of ^137^Cs concentration in the organism, we calculated the ecological half-lives that represents, here, the time (from *T*_*max*_) required for the ^137^Cs activity concentration in the organism to reach the half of its maximum value. The elimination phase of the contamination curve (see Fig 10) is fitted following a one-term exponential model, leading to estimation of the elimination rate. The ecological half-life (***E***) is, afterwards, calculated using the simple relationship $E=\frac{ln2}{\lambda }$. Results of the estimated ecological half-lives are shown in Fig 14 as function of the simulated sites and species. Generally, the species with the highest asymptotic lengths (Tuna species for example) show values higher than those related to the small species. In the case of the small species, the values range between 110 and 300 days with a low spatial variability (coefficient of variation: 4.4% ≤ *CV* ≤ 7.2%). These results are in general consistent with values reported in the previous studies [56,57]. Spatial average of the simulated ecological half-life for the Japanese jack mackerel *Trachurus japonicus* is 215.7±13.0 days, this value matches perfectly the value observed off Fukushima (229±52 days) [56]. However, in the case of the large species the values vary from 360 to about1400 days with a high spatial variability (6% ≤ *CV* ≤ 28%).

**Fig 15:**
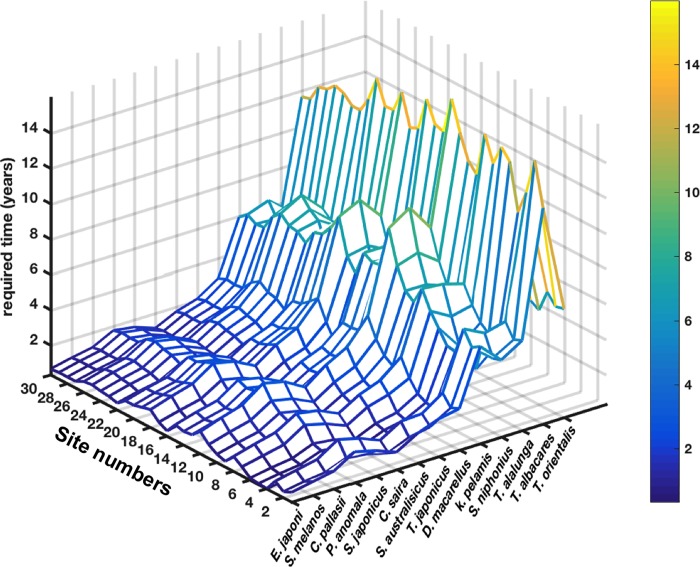
**Estimated time duration (in years) required for the species to reach the pre-accident activity concentrations shown as function of studied species (x-axis) and sites (y-axis).** The studied species are shown in an increasing order of their asymptotic lengths.

From the results of ^137^Cs activity concentration ratios (Fig 13) and those of the ecological half-lives (Fig 14), we estimated the time, in years, required for the studied species to reach the ^137^Cs activity concentrations of the pre-accident steady-state period. Fig 15 shows higher values for the large species compared to the small ones. The estimated values for the small species range between 0.33 and 5.19 years depending upon species and sites. The spatial mean values calculated for these species are ranging between 0.89±0.29 and 3.39±0.77 years. In the case of the large species, the estimated values vary from 3.45 to 16 years, and the spatial mean values lie between 6.36±0.97 and 13.53±1.1 years.

These results mean that the ^137^Cs activity concentrations in the small species have already reached the pre-accident values by 2016 at the latest. While in the case of the large predatory species, even though the activity concentrations are much lower than the regulatory limit for radiocesium in seafood in Japan (100 Bq kg^-1^), they require, on average, a duration of 6–14 years to reach the activity concentrations characterizing the pelagic organisms in the pre-accident period.

## Discussion/Conclusion

### Our model in the context of previous studies

One of the most important challenges in marine radioecology is to estimate the contamination levels of the edible marine species in order to assess the risk related to their contamination and, therefore, to think about the efficient strategies to protect consumers in case of uncontrolled releases of radionuclides in the environment. Modelling is among the important tools being increasingly used by radioecologists to reach these objectives and to document our knowledge on the interactions between the chemistry of these contaminants and the biology (including physiology and ecology) of the organism.

Here we propose a novel modelling approach to simulate the dynamics of radiocesium activity concentration in pelagic marine organisms. The model is afterwards applied to study the contamination levels, by ^137^Cs, of 14 commercially important pelagic species in the Pacific coast of Japan following of the FDNPP accident, which is characterized by a huge contamination of the marine environment. This study has been strongly motivated by two main findings: (1) the lack of more sophisticated radioecological models being able to take into account some biological and environmental factors widely reported in the literature as quite important in the determination of marine organism contamination levels (e.g. the size). (2) The limited nature of the information that can help to understand the mechanism of pelagic species contamination following the FDNPP accident, due to the lack of available data and their low coverage at spatial and temporal scales.

Since it started back to the end of 1950s and the beginning of 1960s, radioecological modelling evolved and became flexible enough to incorporate environmental variability in radionuclide sources, food availability, and organism growth rates in their predictions of organism radionuclide levels [17]. In spite of these progresses, existing radioecological models are still far from proposing a realistic configuration of main processes affecting radionuclide contents in aquatic organisms. The common features of all these models lie in the following points:

- Each species in the model is considered as only one individual with constant size, while in reality, one species is generally composed of many cohorts of different sizes;
- The organism growth rate, which is explicitly taken into account in some models [21,58,59], does not generate any change in their diet. Given that ontogenetic growth is often accompanied with dietary shifts in life history parameters and creates variability in resource use and the strength of trophic interactions [60], this assumption could significantly bias the model results especially in the case of organisms with high growth rate such as the large fish species.
- The diet composition as well as the ingestion rate are considered as constant parameters that are not changing over time, while in reality, these parameters vary in parallel with the organism growth. Indeed, during their life cycle, individuals not only change in size but often also in food requirements, behaviour, trophic interaction, …etc. [61].

It is noteworthy that many previous studies have already highlighted the existing relationship between the ^137^Cs content in the organism and its body size and trophic position. For example, [62] found that ^137^Cs content in brown trout from Wastwater lake after the Chernobyl accident increased with the individual weight. [13] reported that the ^137^Cs activity concentrations in European hake *Merluccius merluccius* from the Mediterranean exhibit an increasing trend with the fish size in relation to change in diet composition. [63] observed the same trend for the silver carp *Hypophthalmichthys molitrix* from the cooling pond of the Chernobyl NPP, while [14] highlighted the presence of a clear positive correlation between mean weight and activity concentration of ^137^Cs in 276 fish samples from the Japanese coasts. [15] and [64] highlighted the year-class-related differences in radiocesium activity concentrations in Pacific cod *Gadus macrocephalus* and Japanese flounder *Paralichthys olivaceus*, respectively, following the FDNPP accident, as result of ontogenetic and spatiotemporal changes in their diet. Other studies highlighted the good correlation between the fish size and the ^137^Cs biological or ecological half-lives (so the ^137^Cs retention), and they proposed, therefore, some allometric relationships connecting this parameter to the organism size [30,65,66].

Our modelling approach is thus proposed in order to complete and improve the existing radioecological models [7,21,22,67]. In this context, our model was developed based on these aforesaid existing models while giving to the organism size a critical role. Our model characteristics can be summarized in the following points:

- Individual-Based model that describes individual organisms as autonomous, unique entities.
- Age-structured model: the species cohorts are explicitly represented in the model. The main parameters affecting the content of ^137^Cs in the marine organism such as the food consumption rate, the diet composition as well as the biological elimination rate are all related to the body size with respect to to the findings reported in previous studies.
- Multi-species: an important number of species can be considered in the model, the interactions between the different species-related individuals are systematically determined by the model according to the predator/prey size ratio under the assumption of opportunistic predation. In our study, we considered 14 species belonging to different levels of the pelagic trophic chain of the Japanese Pacific coast, but it is quite possible, if required, to consider more species if the availability of parameters and computer resources, allow.
- Generic: although this model is until now applied only to study the transfer of one radionuclide, namely ^137^Cs, it is noteworthy that this general modelling approach can be applied to other radionuclides.

In general terms, in addition to the different existing radioecological models, which have already demonstrated their efficiencies in the modelling of radionuclides transfer to the marine biota, our modelling approach is an important achievement for the radioecological modelling by including the organism size as an explicit parameter, allowing one to explicitly represent not only the variability between the different species (inter-species variability), but also among the different cohorts belonging to the same species (intra-species variability). Furthermore, this approach has demonstrated its effectiveness in both accidental and steady state situations, making it a good tool to use for forecasting and risk assessment.

### Current and future situations of the contamination levels in the pelagic species

Application of the model to simulate the ^137^Cs content in 14 commercially important species in the Pacific coast of Japan highlighted the robustness of the model in both steady-state and accidental situations (before and after the accident). The estimated ^137^Cs concentrations were generally in good agreement with the observations in the case of the small species, while in the case of the large species whose the movements are less limited, further field observations are required to validate the results obtained in this study. It should also be noted that in spite of the spatial explicitness of our results, it is wiser that interpretations related to the ^137^Cs content in a given species (especially the large species), be based on the spatial mean at a regional scale rather than on predicted values at a local scale (at a given site) to minimize the biases due to the limited nature of the fish movements assumed in this study.

The results of the simulations clearly point up the return to “normal” conditions (activity concentrations similar to those before the accident) of the majority of small pelagic species about 5 years after the accident (~ from March 2016), and that another 6–14 years would be necessary before seeing the larger species reach the pre-accident levels. This difference is mainly due to the ecological half-life between the species, which is longer in old and larger individuals. In general terms, the dynamics of ^137^Cs transfer along the studied food chain following the FDNPP accident is characterized by a cascading effect due to the gradual trophic interactions. In the first time, the contamination has mainly affected the lower levels of the trophic chain (e.g. sardine, anchovy, herring, …) that are, in general, plankton- and larva-feeding, and which reached their maximum values, in average, 3–4 months after the accident. Afterwards, the higher trophic levels of the trophic chain (feeding on the organisms of the lower levels of the trophic chain) are affected by the contamination, reaching their maximum values 6.5–9 months after the accident. The maximum values reached by all the species were practically similar or slightly higher for the largest species (highest Trophic Levels). However, the rapidity in the contamination process characterizing the species of the lowest trophic levels (small species) compared to the species of the highest trophic levels (large species), is also accompanied by fast elimination process, leading to a more rapid return to the normal conditions in the case of the small species compared to the large ones.

It is important to note that although these results were validated with the observed field data, the forecasts related to this study are carried out under certain assumptions such as the ^137^Cs activity concentrations in seawater that were obtained from dispersion simulations and the limited movement of the individuals, hence the interest to be careful when interpreting these results.

Although the results of this study are, in general, satisfactory, the uncertainty remains high, especially regarding the large species known for their long-distance migratory movements. Consequently, taking into account the fish movement in the radioecological modelling approaches, may greatly improve the predictions especially in the case of an accidental situation with a high radioactive contamination of seawater. Indeed, unlike in a steady-state situation, where the radionuclide activity concentrations are generally spatially homogeneous in both seawater and biota, in an accidental situation, activity concentrations can be very heterogeneous. Consequently, the fish species can, according to their movement trajectories, cross more or less contaminated areas, leading to larger or smaller amounts of accumulated radionuclides. Therefore, despite of its complexity, it is a challenge for the further modelling approaches to explicitly take these movements into account, by coupling an individual based radioecological model with a fish movement model to allow estimation of the radionuclides accumulation in parallel with the fish displacement.

## Supporting information

S1 Fig Two years time series of ^137^Cs concentrations in the 30 sites considered in this study. (PDF)

S1 Table Numerical values of the parameters related to the Von Bertalanffy growth equation and the length-weight relationship used in this study for the 14 studied species. (PDF)

S2 Table Numerical values of the parameters used to estimate the species metabolic rates. (PDF)
