# Supplementary figures and images for: Numerical modelling of 137Cs content in the pelagic species of the Japanese Pacific coast following the Fukushima Dai-ichi Nuclear Power Plant accident using a size-structured food-web model
Source: PLoS One. 2019 Mar 13;14(3):e0212616. doi: 10.1371/journal.pone.0212616 (PMC6415814; doi:10.1371/journal.pone.0212616)

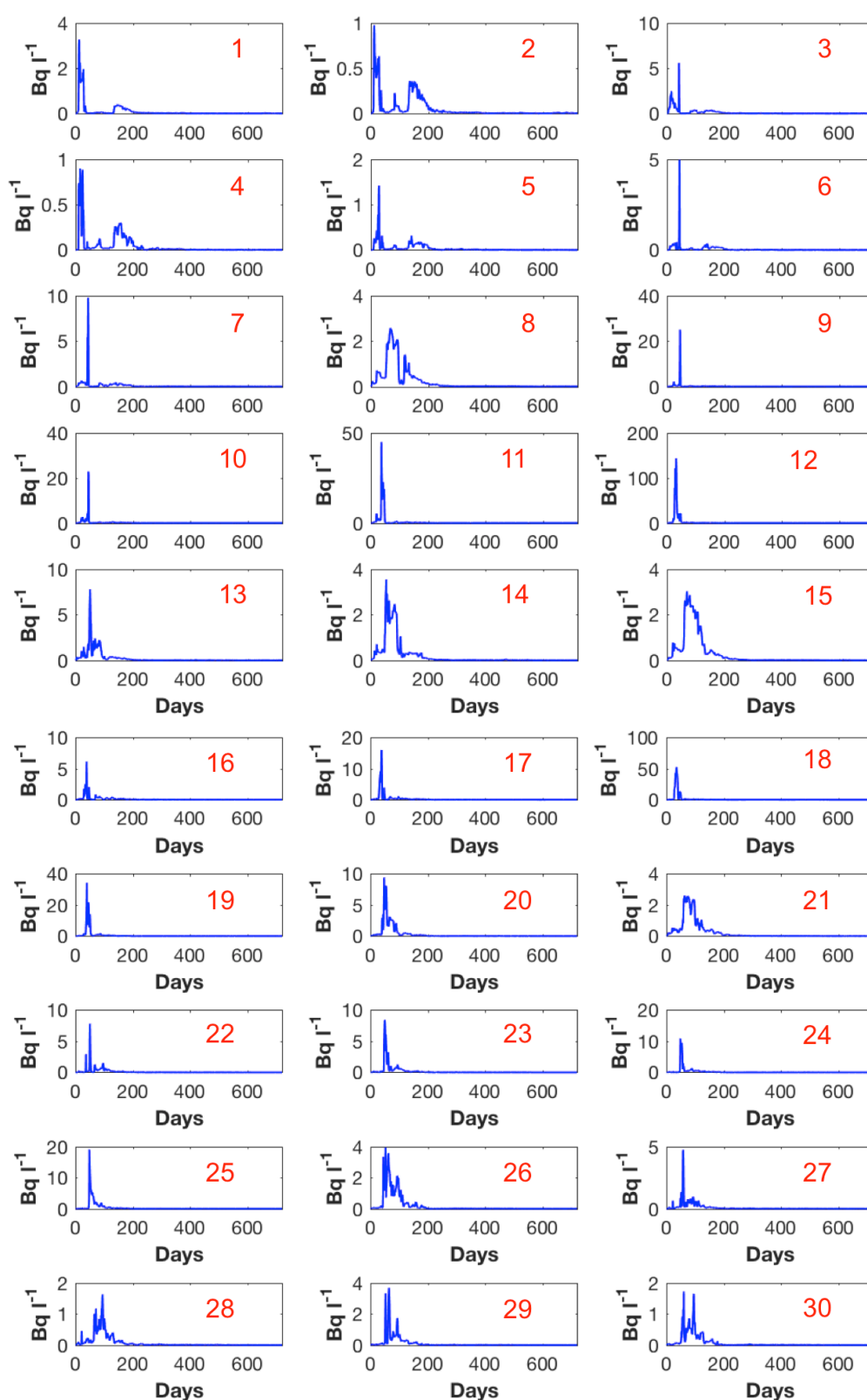

**S1 Figure:** Two years time series of  $^{137}\text{Cs}$  concentrations in the 30 sites considered in this study.

Supplement: S1 Fig — (PDF) [file pone.0212616.s001.pdf]
