# Supplementary material for: Numerical modelling of 137Cs content in the pelagic species of the Japanese Pacific coast following the Fukushima Dai-ichi Nuclear Power Plant accident using a size-structured food-web model
Source: PLoS One. 2019 Mar 13;14(3):e0212616. doi: 10.1371/journal.pone.0212616 (PMC6415814; doi:10.1371/journal.pone.0212616)
Supplement: S1 Table — (PDF) [file pone.0212616.s002.pdf]

Table S1: Numerical values of parameters related to the Von Bertalanffy growth equation and the length-weight relationship used in this study for the 15 fish species. In the case of the cephalopod T. pacificus (last row of the table) the values were obtained by calibration using the equation 7 and the data reported in the literature.

Species	L_∞ (cm)	K (year $^{-1}$)	t_0 (year)	q (g cm^{-b})	b
S. melanostictus	25	0.34	0.51	0.0093	3.06
E. japonicus	20.6	0.94	0.19	0.0047	3.12
C. pallasii	27	0.48	0.35	0.0087	3.04
S. japonicus	33	0.47	0.34	0.0044	3.26
T. japonicus	44.5	0.38	0.39	0.0101	3
D. macarellus	45.5	0.8	0.18	0.0087	3.14
C. saira	36.7	0.38	0.41	0.0023	3.18
P. anomala	28	0.54	0.31	0.0199	3.01
S. australisicus	44.1	0.24	0.63	0.0055	3.22
T. orientalis	320	0.1	0.87	0.0166	3
K. pelamis	79	0.64	0.19	0.0074	3.26
T. albacares	192	0.37	0.27	0.0224	2.94
T. alalunga	159	0.13	0.83	0.0257	2.74
S. niphonius	80	0.91	0.13	0.0077	3
