# Supplementary material for: Numerical modelling of 137Cs content in the pelagic species of the Japanese Pacific coast following the Fukushima Dai-ichi Nuclear Power Plant accident using a size-structured food-web model
Source: PLoS One. 2019 Mar 13;14(3):e0212616. doi: 10.1371/journal.pone.0212616 (PMC6415814; doi:10.1371/journal.pone.0212616)
Supplement: S2 Table — (PDF) [file pone.0212616.s003.pdf]

S2 Table: Numerical values of the parameters used to estimate the species metabolic rates.

**Species**	a_R	b_R	c_R	d_R
S. melanostictus	0.1375	0.77	0.03	0.03
E. japonicus	0.1375	0.77	0.03	0.03
C. pallasii	0.1375	0.77	0.03	0.03
S. japonicus	0.1833	0.77	0.03	0.03
T. japonicus	0.1375	0.77	0.03	0.03
D. macarellus	0.1833	0.77	0.03	0.03
C. saira	0.1375	0.77	0.03	0.03
P. anomala	0.1408	0.8	0.03	0.03
S. australisicus	0.1833	0.77	0.03	0.03
T. orientalis	0.1408	0.75	0.085	0.01
K. pelamis	0.1408	0.75	0.085	0.01
T. albacares	0.1408	0.75	0.085	0.01
T. alalunga	0.1408	0.75	0.085	0.01
S. niphonius	0.1833	0.77	0.03	0.02
